# A wild Indo-Pacific bottlenose dolphin adopts a socially and genetically distant neonate

**DOI:** 10.1038/srep23902

**Published:** 2016-04-06

**Authors:** Mai Sakai, Yuki F. Kita, Kazunobu Kogi, Masanori Shinohara, Tadamichi Morisaka, Takashi Shiina, Miho Inoue-Murayama

**Affiliations:** 1 Department of Fisheries, Kindai University, 3327-204 Nakamachi, Nara, 631-8505, Japan; 2 Department of Marine Biology and Sciences School of Biological Sciences, Tokai University, 5-1-1 Minamisawa, Minami-ku, Sapporo-shi, Hokkaido 005-8601, Japan; 3 Mikura Island Tourist Information Centre, Mikurajima-mura, Tokyo 100-1301, Japan; 4 Faculty of Life and Environmental Sciences, Teikyo University of Science, 2525 Yatsusawa, Uenohara-shi, Yamanashi 409-0193, Japan; 5 Institute of Innovative Science and Technology, Tokai University, 3-20-1 Orido, Shimizu-ku, Shizuoka-shi, Shizuoka 424-8610, Japan; 6 Department of Molecular Life Science, Division of Basic Medical Science and Molecular Medicine, Tokai University School of Medicine, 143 Shimokasuya, Isehara-shi, Kanagawa 259-1143, Japan; 7 Wildlife Research Center, Kyoto University, 2-24 Tanaka-sekiden-cho, Sakyo-ku, Kyoto-shi, Kyoto 606-8203, Japan; 8 Wildlife Genome Collaborative Research Group, National Institute for Environmental Studies, 16-2 Onogawa, Tsukuba-shi, Ibaraki 305-8506, Japan

## Abstract

Alloparental behaviour and adoption have been reported in many mammals and birds. Such behaviours are energetically costly, and their causes and functions remain unclear. We observed the adoption behaviour of a wild Indo-Pacific bottlenose dolphin (*Tursiops aduncus*) near Mikura Island, Japan. A calf was seen with its mother on six observation days. Following the mother’s death, the calf was observed with a sub-adult female on all 18 observation days from May to September 2012. On three days, the calf was observed swimming with this female in the suckling position and milk was seen leaking from the female’s mammary slit. A five-year dataset revealed no significant social or kin relationships between the biological mother and allomother, indicating that kinship and social relationships did not play an important role in the observed adoption.

Associations and/or interactions between infants and non-parents, termed as alloparental behaviour, have been reported in many mammals^1^,^2^,^3^. However, it is not always clear whether this behaviour involves alloparental care for the infant. Hrdy^4^ classified allomaternal behaviour according to ‘the sort of behaviour at issue, be it allomaternal care, abuse, or indifference.’ Mann *et al*.^5^ suggested, ‘allomaternal behaviour can vary considerably, both within and between species, in the form that it takes and the consequences that it has for infants, mothers, and allomothers’.Allomaternal behaviour has been reported in captive and wild cetaceans such as bottlenose dolphins (*Tursiops* sp.)^5^,^6^,^7^,^8^, sperm whales (*Physeter macrocephalus*)^9^,^10^,^11^, spinner dolphins (*Stenella longirostris*)^12^, killer whales (*Orcinus orca*)^13^, harbour porpoises (*Phocoena phocoena*)^14^, Atlantic white-sided dolphins (*Lagenorhynchus acutus*)^15^, and belugas (*Delphinapterus leucas*)^16^. Sperm whale allomothers provide vigilant watch of calves at the surface, while mothers make deep dives for food^10^,^11^ and some allomothers nurse the young^10^. Mann *et al*.^5^ observed that wild bottlenose dolphin escorts (allomothers) did not benefit mothers by allowing them to forage. In our study area, we have often observed infants swimming with alloparents, but the function of this behaviour remains unclear.

Adoption, a form of alloparental behaviour, occurs in the absence of one or both genetic parents. In assuming the role of a foster parent, the animal provides exclusive care for the offspring of another^2^. Adoption has been reported in every major mammalian taxon^2^. The behaviour is costly for foster parents, and it is unclear why an animal would invest its resources in this manner. Several hypotheses have been proposed^2^, but controversy remains. Previous studies have reported adoption and found that parous female bottlenose dolphins in several reproductive stages (lactating, non-lactating, pregnant or non-pregnant) can lactate and feed unrelated calves^17^,^18^,^19^,^20^. However, it remains unknown whether nulliparous dolphins can lactate. In the natural environment, there has been only one report of an adult female bottlenose dolphin associating with an orphan^21^. To our knowledge, there is no report on the direct care of an orphaned cetacean by an allomother or information about the relationship between biological mothers and foster mothers in the wild.

Here, we report on the adoption behaviour of a wild Indo-Pacific bottlenose dolphin, as well as the social and kin relationships between the biological mother and the foster mother. We also discuss the socio-behavioural context for adoption behaviour.

## Results

### Process of adoption

The biological mother (dolphin #356) was a parous female, 15 years old in 2012, and had a slightly developed abdomen in September 2011. The adoptive mother (dolphin #576) was a sub-adult female, 8 years old in 2012. The adoptive mother was never observed continuously associating with a calf; therefore, we assumed that she was either nulliparous or had never experienced reproductive success.

**Figure 1:**
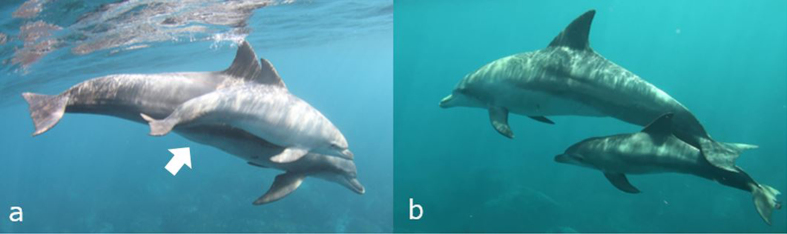
A male calf and his associates. (**a**) The calf with #356 in the echelon position (16 May, photographed by Nana Takanawa). (**b**) The calf with #576 in the infant position (3 July, photographed by Mai Sakai).

On all six days that dolphin #356 was observed, a male calf was observed with her in the infant position (Table 1). Infant bottlenose dolphins are seldom observed in the infant position with non-mothers^5^, and #356 had well-developed mammary glands when it was first observed with a male calf on 27 April 2012 (Fig. 1a). Therefore, we concluded that #356 was the biological mother of the calf. The foetal lines of the calf were visible at the initial observation and became indiscernible by 21 August. Foetal lines become indiscernible by month three^7^; therefore, we estimated that it was born in April 2012. On 25 May 2012, the mother was found dead, entangled in a recreational fishing net. During the autopsy, we collected a skin sample for genetic analysis. A foster mother (#576) was first observed with the calf on 9 June 2012 and was observed with the calf for 18 days until 19 September. The foster mother was monitored regularly after that date, but the calf was not observed with it.

The calf was observed taking the infant position only when associating with its mother or foster mother (Table 1). The calf was observed in the suckling position with the foster mother. On 3 July, we observed milk leaking from the mammary slit of the foster mother after the calf removed its rostrum. The calf was observed taking the echelon position and other positions when associating with other individuals (Table 1). The calf appeared well fed during the time he was observed with his mother (Fig. 1a). However, he appeared thinner than other calves when he associated with the foster mother (Fig. 1b).

### Behavioural observations over 5 years

The mother and foster mother were concurrently observed at 16 observation sequences from 2007 to 2011. Social interactions (e.g. physical contact, parallel swimming, and synchronous breathing) between them were never observed during these observation sequences. We calculated the association indices (half-weight indices; HWI) between the mother and foster mother for each year from 2007 to 2011. There was no evidence of any significant or strongly preferred association or avoidance between them over five years (see methods, Table 2).

### Analysis of genetic relatedness

Genetic samples were collected from 54 identified individuals between 2010 and 2012. Tests for linkage disequilibrium demonstrated independent assortment of the 12 loci. Levels of genetic variation and frequencies estimated using CERVUS^22^ are shown in Supplementary Tables S1 and S2. Sequence analysis of 415 or 416 bp of the mitochondrial DNA control region for these 54 dolphins identified four haplotypes (JTa01, JTa02, JTt10 and TT037; GenBank accession numbers: LC003514, LC003515, AB303163 and HQ436284, respectively). The most frequent haplotype was JTa01 at 0.778 (Supplementary Table S1).

**Figure 2:**
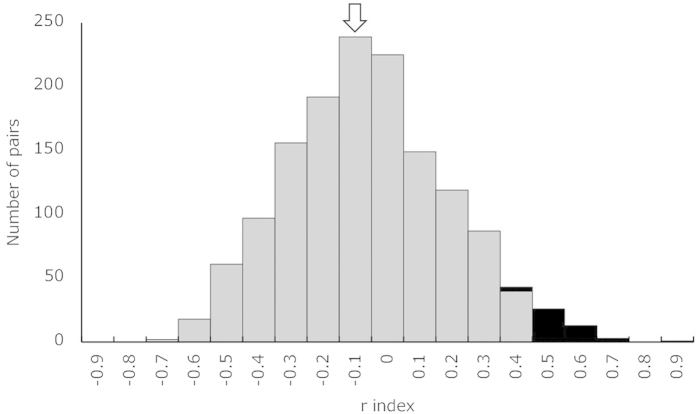
Relatedness indices for pairwise combinations of dolphins in the study population. Black bars suggest a kinship. The arrow indicates the bar including the *r* index for the biological mother and foster mother.

The relatedness indices for pairwise combinations for the 54 individuals ranged from −0.695 to 0.912 (mean ± SE = −0.010 ± 0.007, 95% confidence interval −0.502 to 0.483). Pairs with an *r* index >0.483 were considered to have a kin relationship (Fig. 2). The *r* index of the biological mother and foster mother was −0.086, lower than the average *r* index.

## Discussion

Following the biological mother’s death, the calf associated almost exclusively with the foster mother and this relationship lasted for 102 days (Table 1). Non-mother Indo-Pacific bottlenose dolphins have frequently been observed swimming with calves^5^, but there are no previous reports of allomaternal nursing in this species^5^. Our observations suggest that the foster mother built a relationship with the calf that differed from the typical allomother-calf relationship, suggesting adoption of the calf by the foster mother.

It should be noted that the foster mother nursed the calf although she was probably nulliparous. This is significant because adoptions are generally undertaken by females with infants or in pregnant females^2^,^3^, but rarely in nulliparous females. Induced lactation, in which milk is produced in the absence of a previous pregnancy or lactation, has been reported in humans^23^,^24^ and several domestic animals^25^,^26^,^27^, but less frequently in cetaceans. Ridgeway (1995)^19^ reported that a probable non-pregnant female was observed nursing an orphan. Parous females have been observed nursing orphans in captive bottlenose dolphins^17^,^19^,^20^.

It is possible there are differences in the social and physical development between orphaned calves and those raised by their biological mother. As they increase in age bottlenose dolphin calves spend more time separated from their mothers and increase the number of associates^28^. In this study, the calf associated with three females while the mother was alive, but associated with only one sub-adult other than the foster mother following the biological mother’s death. We observed flipper rubbing behaviour between the foster mother and calf on five observation days, with the pair taking turns both providing and receiving rubbing. This was an exceptional case because infant dolphins seldom act as the provider during flipper rubbing behaviour^6^,^29^. This suggests some socio-behavioural differences between the biological mother-calf pair and foster mother-calf pair. The calf became thinner when it associated with its foster mother (Fig. 1), indicating that the foster mother’s care might not have been sufficient to sustain the calf.

Individuals that care for or nurse alien young may acquire selective advantages associated with a number of factors^2^,^3^. We rejected the possibility that the foster mother increased its inclusive fitness by caring for related calves because the biological mother and the foster mother were genetically distinct. Direct social interactions between them were not observed. They also did not share many observation sequences. These results suggest that this adoption was not caused by preferred social relationships between the biological mother and foster mother, or the calf and foster mother. Therefore, #576 (the foster mother) may not have actively chosen to adopt the orphan of #356 (the biological mother) and that the orphan calf initiated the behaviours that resulted in the foster mother adopting the calf. In one study on captive bottlenose dolphins, orphans immediately tried to nurse from allomothers, and dry adult dolphin females were brought into lactation by repeated nursing attempts^19^. In the current study, it was easy for the foster mother to refuse and/or escape from the calf, but it did not. Several helping behaviours have been reported in dolphins^14^,^30^,^31^,^32^. These behaviours suggest that dolphins have the capacity for empathic perspective-taking^33^, but the precise nature of these cognitive abilities is unknown. The cognitive characteristics that evoke adoption behaviours in dolphins need additional study to determine the extent to which they reflect social cognition or more generic responses to the behaviour of individuals in need.

## Methods

### Study area and study period

The study was conducted at Mikura Island (33°53′N, 139°36′E; approximately 16 km of coastline). All observations were conducted in a 300 m area offshore, at depths of 2–45 m during spring and autumn from 2007 to 2012.

### Dolphin population

Behaviours were observed by underwater video recordings. Each dolphin was identified by natural markings on the body. Videos were recorded by members of the photo-identification research team of the Tourist Information Centre of Mikurashima Island (TICM). We also used underwater photos and videos taken by tourists and guides during swimming with dolphin tours (see Acknowledgements). The size of the study population of Indo-Pacific bottlenose dolphins was estimated at about 160^34^ (TICM, unpublished data). The number of dolphins varied between 2008 and 2012 (approx. 140 in 2008 and 2009, 130 in 2010, 110 in 2011 and 120 in 2012; TICM, unpublished data). The sex of the dolphins was determined by examining the genital slit. We classified dolphins into four age classes: adult, sub-adult, juvenile, and neonate^34^. Neonates were considered to have been born in the year when first identified. A mother-calf pair was defined as a pair comprised of an adult female and calf observed together for more than 50% of the total observations of an adult female^34^.

### Behavioural data and genetic sample collection

Once a school was detected, a researcher entered the water and recorded dolphin behaviour using a digital camcorder in a waterproof housing (Sony Corp., Japan or NTF Corp., Japan). An *ad-lib* protocol was adopted for sampling^35^. After the school passed, the researcher returned to the boat. The boat then approached the same school again or searched for another school. Each research trip lasted approximately two hours. We collected faeces samples for genetic analysis. When a dolphin defecated during an underwater observation, we collected it using a plastic tube and used video-recorded or direct observations to identify the individual.

### Ethics statement

To minimize disturbance, we followed a non-invasive approach for observation. In most cases, dolphins did not show any unusual behaviour during observations. We did not use scuba tanks, we never attempted to touch the dolphins, and we never fed them. This study was conducted in accordance with the recommendations of the Guidelines to Study Wild Animals of the Wildlife Research Center of Kyoto University and the voluntary regulatory rule for sustainable dolphin swimming programs developed by the program operators on Mikura Island. Permission to enter the protected sea area around Mikura Island was granted by Mikurashima village. All research protocols were approved by Mikura Island Tourist Information Centre.

### Observation sequence

**Figure 3:**
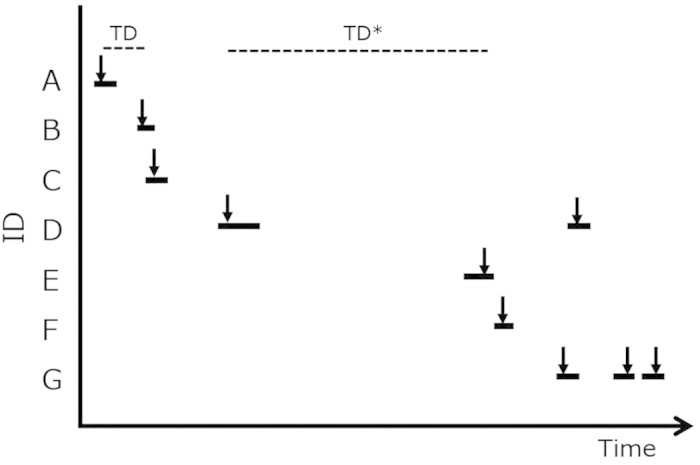
The definition of the observation sequence. Black bars indicate the segment of a video recording from when a dolphin appeared in the video until it left the frame. Arrows indicate the best time image from a video recording. TD indicates the time difference between nearest best time images. *indicates TD > 16.3 min. There were two observation sequences, the former sequence includes A, B, C and D, and the latter includes D, E, F and G.

**Figure 4:**
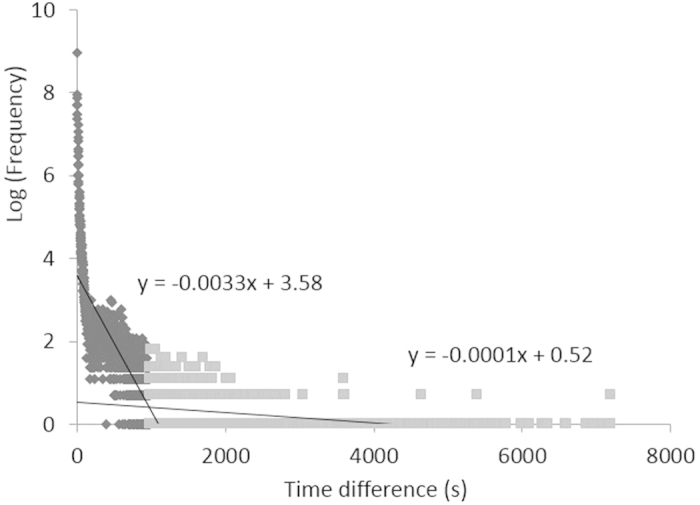
Log frequency of TD used to define the observation sequence. Logarithms of actual frequencies of TD length were plotted against TD lengths and approximated to a broken-stick curve. Black dots represent the group with shorter TD lengths (<16.3 min), and grey dots represent the group with longer TD lengths (>16.3 min).

Bottlenose dolphins (*Tursiops* sp.) show a fission-fusion grouping pattern in which individuals associated in small groups that changed in composition, often on a daily or hourly basis^36^. In this study area, dolphins often changed their group composition and/or spatial distance among individuals. We defined an observation sequence as the period when several dolphins were observed continuously using underwater video data instead of defining groups. We recorded the ‘best time’ images of each dolphin, defined as the time when the dolphin was close to the video camera and was easy to identify. We calculated time differences (TD) between each nearest best time (Fig. 3). A broken-stick model was used to divide TD from 0 to 7200 s into two groups. We excluded TD > 7200 s from this analysis because one research trip lasted approximately 2 hours. The residual sum of squares was smallest when the samples were divided into two groups, one with a TD of 0–977 s (16.3 min) and one with a TD of 978–7200 s (Fig. 4). Dolphins with shorter TD lengths (<16.3 min) were considered to be in the same observation sequence. Dolphins with longer TD lengths were not considered to be in the same observation sequence. We defined an observation sequence using a 16.3-minute chain rule in which any animal with a best time within 16.3 minutes of the best time of any other animal was considered to be in a same observation sequence (Fig. 3). Two dolphins have the chance to conduct social interactions if they shared some observation sequences.

### Association analysis

We used the half-weight index (HWI) = 2X/[A + B], where X is the number of times dolphins A and B were seen in same observation sequence, and A and B are the total number of times dolphins A and B were sighted, for calculating association strength^37^. Individuals were only used in the analysis if they had been observed on more than four days during each year^38^. Preferred associations, i.e. associations occurring more often than expected by chance, in each year were defined using the Manly Bejder permutation technique^39^. This technique tests the significance of these associations by randomly permuting individuals within observation sequences, keeping the number of individuals sharing same observation sequence and the number of times each individual was observed constant. The analysis was performed using SOCPROG compiled version 2.4^40^ and was achieved by switching two individuals present in two different observation sequences. After each permutation, the HWI for each pair was calculated and the observed HWI was compared with expected values of the HWI. If the real HWI was >97.5% of the random HWI, the relationship between the pair of dolphins was defined as a preferred association. The observed association matrix was randomized 100,000 times with 1,000 trials per permutation. We assessed association strength between the mother (#356) and the allomother (#576) in each year from 2007 to 2011, when they were sub-adult or adult.

### Behavioural analysis

We recorded the spatial relationships between the calf and its nearest neighbours. The swimming position was classified into the following four types: echelon position (the calf is roughly parallel, touching the other dolphin’s flank above the midline^6^), infant position (the calf swims under the other dolphin, the melon or head lightly touches the other’s abdomen^6^), suckling position (the calf touches its rostrum to the mammary silt of the other dolphin) and other position (a position other than one of the three aforementioned positions). One-zero sampling^35^ was used to record the positions with each associate. We determined the four positions each observation day by checking photos and/or video recordings. Non-agonistic social interactions, in which flipper rubbing, synchronous breathing and parallel swimming were analysed using video data.

### Genetic analysis

Faeces samples were preserved in 99.5% ethanol in a cold storage chamber. Total genomic DNA was isolated from faeces using the QIAamp DNA Stool Mini Kit (Qiagen, Germany) according to the manufacturer’s protocol. Two sets of genetic markers were used to test for kin relationships among individuals: 12 nuclear DNA microsatellite loci markers (DlrFCB4, DlrFCB16^41^, EV5^42^, KWM9b, KWM12a^43^, MK3, MK5, MK6, Mk8, MK9^44^, TexVet5, and TexVet7^45^) and a 415–416-bp fragment of e mitochondrial DNA control region, which was amplified using D-loop primer^46^. These genetic markers were analysed using the protocol described by Kita *et al*.^46^.

Pairwise kin relationships of 1431 pairs among the 54 dolphins analysed at 12 microsatellite loci were estimated using the relatedness coefficient index, *r*^47^. The index was calculated as follows [Equation (1)].


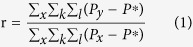


where *x* represents individuals in the data set, *k* represents loci, *l* represents allelic position (i.e. *l* = 1 or 2 for a diploid individual) and *P*_*x*_ is the frequency within the current individual *x* of the allele found at *x*’s locus *k* and allelic position *l*; this value in a diploid must be either 0.5 or 1.0. *P*_*y*_ is the frequency of the same allele in the set of ‘partners’ of *x* in the individual(s) being investigated for relatedness to *x*. P* is the frequency of the allele in the population at large. The coefficient *r* was calculated by comparing the alleles shared by two individuals with the frequency of that allele in 54 individuals. Calculations were performed using the program RELATEDNESS v5.0.8 (K. F. Goodnight, Rice University, Houston, TX, USA). Average genetic relatedness and standard errors were obtained by jackknifing over all loci^47^.

## Additional Information

**How to cite this article**: Sakai, M. *et al*. A wild Indo-Pacific bottlenose dolphin adopts a socially and genetically distant neonate. *Sci. Rep*. **6**, 23902; doi: 10.1038/srep23902 (2016).

## Supplementary Material

Supplementary Information

## Figures and Tables

**Table 1:** Observation days and the spatial positions of a male calf and his associates.

Month	Day	Associates	EP	IP	SP	Other
April	27	#356FA	X			
May	7	#356FA	X			X
		#068FA	X			
	8	#356FA		X		
	14	#356FA	X	X		
		#601FS	X			X
	16	#356FA	X			
		#507FS	X			X
	17	#356FA	X	X		
		#507FS	X			
	25	*#356 was found dead*				
June	9	#576FS	X			X
	28	#576FS		X	X	
July	1	#576FS	X			X
	2	#576FS	X	X		X
	3	#576FS	X	X	X	X
		Unknown S	X			
	9	#576FS	X	X		X
	13	#576FS		X		
	20	#576FS		X	X	
	26	#576FS		X		
August	2	#576FS	X			
	18	#576FS	X	X		
	21	#576FS	X			
	31	#576FS		X		
September	2	#576FS	X	X		
	4	#576FS	X			
	6	#576FS	X			X
	7	#576FS				X
	19	#576FS		X		

Abbreviations. EP, echelon position; IP, infant position; SP, suckling position; Other, other position; FA, adult female; FS, sub-adult female; S, sub-adult. X indicates the positions observed on each observation day.

**Table 2:** Association indices of #356 and #576, and parameters of HWI for all associations in the study population.

Year	No. of individuals analysed	HWI of #356 and #576	Parameters of HWI for all associations			
			mean	SD	CV	Proportion of non-zero elements
2007	146	**0.34**	**0.12**	**0.10**	**0.86**	**0.77**
			0.12	0.09	0.73	0.80
2008	130	**0.14**	**0.12**	**0.11**	**0.94**	**0.70**
			0.12	0.10	0.81	0.74
2009	134	**0.17**	**0.12**	**0.12**	**1.04**	**0.65**
			0.12	0.10	0.83	0.72
2010	114	**0.24**	**0.15**	**0.12**	**0.78**	**0.82**
			0.15	0.10	0.69	0.83
2011	89	**0.11**	**0.12**	**0.12**	**0.97**	**0.68**
			0.12	0.10	0.83	0.73

Individuals were only included in the analysis if they had been observed on more than four different days during a year. Underlined figures show the HWI calculated from randomised data. Bold figures show the HWI calculated from real data.
