# Supplementary material for: A wild Indo-Pacific bottlenose dolphin adopts a socially and genetically distant neonate
Source: Sci Rep. 2016 Apr 6;6:23902. doi: 10.1038/srep23902 (PMC4822121; doi:10.1038/srep23902)
Supplement: Supplementary Information [file srep23902-s1.pdf]

A wild Indo-Pacific bottlenose dolphin adopts a socially and genetically distant neonate

Mai Sakai[1,2*], Yuki F. Kita[3], Kazunobu Kogi[4], Masanori Shinohara[5], Tadamichi Morisaka[1], Takashi Shiina[6] and Miho Inoue-Murayama[7,8]

Supplemental Information

Supplementary Table S1. Allele frequency of 12 microsatellite markers and mitochondrial DNA D-loop region genotypes for 54 dolphins. Abbreviations. A.F, allele frequency; G.F, genotype frequency. Bold letters show allele and genotype frequencies >0.1.

DlrFCB4		DlrFCB16		TexVet5		TexVet7		EV5		KWM9b		KWM12a	
allele	A.F.	allele	A.F.	allele	A.F.	allele	A.F.	allele	A.F.	allele	A.F.	allele	A.F.
**1**	**0.157**	**3**	**0.306**	1	0.009	1	0.009	**1**	**0.148**	1	0.009	**1**	**0.148**
4	0.009	**4**	**0.556**	**3**	**0.352**	**3**	**0.426**	**2**	**0.824**	2	0.083	2	0.019
5	0.083	**5**	**0.111**	**4**	**0.593**	**4**	**0.148**	3	0.028	**5**	**0.407**	**3**	**0.194**
6	0.009	6	0.019	5	0.04	**5**	**0.380**			6	0.056	4	0.009
**7**	**0.722**	7	0.009	6	0.01	7	0.037			**7**	**0.259**	**6**	**0.611**
9	0.009									8	0.083	7	0.009
13	0.009									9	0.083	8	0.009
										10	0.009		
										13	0.009		
7 alleles		5 alleles		5 alleles		5 alleles		3 alleles		9 alleles		7 alleles	

Mk3		Mk5		Mk6		Mk8		Mk9		mtDNA D-loop	
allele	A.F.	allele	A.F.	allele	A.F.	allele	A.F.	allele	A.F.	Haplotype	G.F.
1	0.009	**1**	**0.426**	1	0.009	**1**	**0.259**	1	0.009	JTa01	**0.778**
**2**	**0.444**	**2**	**0.417**	**2**	**0.620**	2	0.093	**6**	**0.463**	JTa02	0.093
3	0.037	**3**	**0.120**	**3**	**0.185**	3	0.019	**7**	**0.250**	TT037	**0.111**
**4**	**0.148**	4	0.028	**4**	**0.148**	**5**	**0.630**	8	0.028	JTt10	0.019
5	0.009	5	0.009	11	0.037			**10**	**0.250**		
**6**	**0.352**										
6 alleles		5 alleles		5 alleles		4 alleles		5 alleles		19 alleles	

Supplementary Table S2. Statistical results for the genetic analysis of 12 MS markers of 54 dolphins. H (O) and H (E) indicate observed and expected heterozygosities, respectively. HWE indicates Hardy–Weinberg's equilibrium (P values).

Locus	Allele Num.	H (O)	H (E)	HWE
DlrFCB4	7	0.407	0.451	0.910
DlrFCB16	5	0.519	0.591	0.882
TexVet5	5	0.407	0.528	0.932
TexVet7	5	0.648	0.657	0.668
EV5	3	0.111	0.301	0.991
KWM9	9	0.796	0.750	0.313
KWM12	7	0.444	0.571	0.973
Mk3	6	0.611	0.661	0.805
Mk5	5	0.611	0.636	0.697
Mk6	5	0.574	0.563	0.485
MK8	4	0.500	0.532	0.920
Mk9	5	0.611	0.666	0.859
